# Strategies to improve the quality of wheat- flour- bread chain in Iran: the perspective of different stakeholders

**DOI:** 10.1186/s13104-022-06225-7

**Published:** 2022-10-22

**Authors:** Marjan Bazhan, Farnam Shafiei Sabet

**Affiliations:** 1 Department of Community Nutrition, Faculty of Nutrition Sciences and Food Technology, National Nutrition and Food Technology Research Institute, Shahid Beheshti University of Medical Sciences, Tehran, Iran; 2 Department of Food Science and Technology, Faculty of Nutrition Sciences and Food Technology, National Nutrition and Food Technology Research Institute, Shahid Beheshti University of Medical Sciences, Tehran, Iran

**Keywords:** Quality improvement, Wheat- flour- bread chain, Qualitative study

## Abstract

**Objective:**

Bread constitutes a significant energy source and provides protein and some essential micronutrients to a large population worldwide, including Iran. So, its quality characteristics are important for health. This study aimed to identify the views and experiences of various stakeholders involved in the wheat- flour- bread chain about factors affecting the quality of this chain and strategies for its improvement in Iran.

**Results:**

Main suggested strategies in the field of raw materials were managing and planning for the production of high-quality wheat, measuring the quality factors of grain before purchasing by the government, allocating wheat quotas to flour mills based on the quality of the flour produced, and aerating and storing flour in silos to reduce its moisture. Holding training courses for bakery workers, improving the economic situation of bakers, and standardizing bread-making devices were the most important strategies in the bakery field. Assigning a specific unified management apparatus to deal with bread issues was also an effective and essential strategy. Findings show the need to implement strategies in various fields to improve wheat- flour- bread chain quality. This study provides helpful information to guide policy decisions and planning to enhance bread quality and promote public health.

**Supplementary Information:**

The online version contains supplementary material available at 10.1186/s13104-022-06225-7.

## Introduction

Bread is the most important wheat-based staple food worldwide [1, 2], including in Iran [3]. Iranians are the second biggest bread consumers in the world after Turkish consumers. The average daily bread consumption in Iran is about 320 g, up to nearly 117 kg per capita annually [4], almost twice that of European countries [5]. There are many factors involved in reducing the quality of bread and increasing its waste, including the quality of the raw material [6–8], the process of making bread [7], the knowledge and attitude of bakers and consumers about baking, consuming, and storing bread [9, 10]. The bread quality plays an essential role in people’s health and the national economy, and improving its situation requires serious attention. The factors affecting bread quality and strategies for improving its quality have not been comprehensively studied in Iran. So, this study was carried out to identify the views and experiences of various stakeholders involved in the wheat- flour- bread chain about factors affecting bread quality and strategies for its improvement.

## Main text

### Study design and data collection

This qualitative research consisted of 34 in-depth interviews with key stakeholders related to the wheat- flour- bread chain and ten focus groups (FGs) with consumers in Tehran, the capital of Iran (Table 1). Purposive and snowball sampling was used to select participants. The criteria for selecting participants were the willingness and ability to share their opinions and experiences.

Office correspondence was made with the relevant organizations to arrange the interviews with informed and experienced persons in the field of study to collect data from key stakeholders (except for traditional bread bakers). Concerning traditional bread bakers, sampling was done in such a way as to cover all geographical regions of the city. So, one district was randomly selected from each geographical region. Then, four bakeries (sangak/ barbary/ taftoon/ and lavash bakery) were randomly chosen in that district. The person most involved in making bread in each bakery was interviewed. FGs were separately conducted for housewives recruited from local community centers belonging to Tehran Municipality’s Social and Cultural Department and employed women/ men recruited from government organizations. Like the bakers, sampling was done from different geographical regions of Tehran to ensure sample diversity. Individuals interested in participating in the study were invited to join study.

All interviews were conducted in a separate room using a semi-structured guide (Additional file 1) by the main researcher, and a second researcher took notes. The individual interviews and FGs were audiotaped with the participants’ permission, transcribed verbatim, and analyzed consecutively in Farsi. More details are provided in Additional file 2.

### Data analysis

Transcripts of all interviews were imported into MAXQDA®, a software package for managing and analyzing qualitative data. Directed thematic analysis was used to analyze the data [11].

**Table 1:** Stakeholder groups participating in the study (n = 117)

Stakeholder groups	Subgroups of stakeholders	n
Stakeholders in the wheat and flour production and supply field	- Ministry of Agriculture- Jihad (Vice- Dean in Agriculture Affairs, and Agricultural Research, Education & Extension Organization) **-** Ministry of Industry, Mine, & Trade (Grain Research Center- Government Trading Corporation of Iran) **-** Seed & Plant Improvement Institute **-** Knowledge-Industry-Market Coordinating Center of Wheat- Flour- Bread Chain - Iranian Association of Flour Milling Industries	8
Stakeholders in the standardization and quality control of wheat- flour- bread chain	- Iranian National Standards Organization - Ministry of Health & Medical Education (Community Nutrition Improvement Office, Food and Drug Administration, and Center for Environmental and Occupational Health)	3
Stakeholders in the bread-making and supply field	- Tehran Traditional Bread Bakers’ Union - Sangak bread Bakers’ Union - Traditional bread bakers	23
Consumer stakeholders	- Adult men and women living in Tehran, the capital of Iran	83

## Results

The characteristics of the study participants are shown in Table 2. Five strategies to improve bread quality emerged from the data analysis (Table 3), which are explained in detail below.

**Table 2:** The study participants’ characteristics

Participants’ characteristics of individual interviews						
	Stakeholders in the wheat and flour production & supply field (n = 8)	Stakeholders in the standardization & quality control of wheat- flour- bread chain (n = 3)			Stakeholders in the breadmaking & supply field (n = 23)	
Age (year) ^*^	45.4 (11.2)	47.0 (7.6)			47.8 (9.7)	
Sex^**^ Women						
	3 (37.5)	2 (66.6)			-	
Men	5 (62.5)	1 (33.4)			23 (100.0)	
Education^**^						
Under diploma	-	-			16 (69.6)	
Diploma	-	-			7 (30.4)	
University	8 (100.0)	3 (100.0)			-	
Work experience^*^	16.3 (9.2)	20.0 (5.6)			29.0 (13.8)	
**Participants’ characteristics of focus group interviews**						
			Housewives (n = 40)	Employed women (n = 23)		Men (n = 20)
			N (%)	N (%)		N (%)
Age (year)						
25–34			11 (27.5)	7 (30.4)		1 (5.0)
35–44			15 (37.5)	8 (34.8)		10 (50.0)
45–54			6 (15.0)	4 (17.4)		1 (5.0)
55^+^			8 (20.0)	4 (17.4)		8 (40.0)
Education Under diploma						
			16 (40.0)	-		-
Diploma			8 (20.0)	-		-
University			16 (40.0)	23 (100.0)		20 (100.0)
Marital status						
Married			32 (80.0)	19 (82.6)		15 (75.0)
Unmarried			8 (20.0)	4 (17.4)		5 (25.0)
Responsible for buying bread in the household						
Yes, always			24 (60.0)	6 (26.1)		13 (65.0)
Yes, sometimes			12 (30.0)	14 (60.9)		4 (20.0)
No			4 (10.0)	3 (13.0)		3 (15.0)

^*^ Mean (SD), ^**^ N (%)

**Table 3:** Suggested strategies to improve the quality of wheat- flour- bread chain from the perspective of various stakeholders

Categories	Codes	
Strategies in the field of wheat	- Managing and planning for producing high-quality wheat - Industrialization of the agricultural system - Observance of the correct principles of wheat planting and harvesting - Mandatory implementation of the wheat standard - Measuring the quality factors of wheat before purchasing - Transferring the purchase of wheat to the non-governmental sector - Separating wheat in terms of quality in factories - Mixing different wheat varieties to get good quality flour - Storing wheat in silos for a certain time to improve its quality - Importing wheat from abroad and mixing it with domestic wheat - Strengthening and expanding cooperatives to buy and sell wheat	*“Why did wheat imports stop? Let it be imported and mixed with domestically produced wheat. When the imports are stopped, how is it possible to compete in the market with such poor-quality wheat? Can we bake high-quality bread? Of course not. No baker likes to sell poor-quality bread to people” (One of the bakers)*. *“We have different varieties of wheat that, if not mixed, we won’t have high-quality wheat. Each wheat variety will have the best quality if cultivated in a specific season. Different wheat varieties also have different colors. If they are properly mixed, the resulting flour will have a good color” (Head of traditional bread bakers’ union).*
Strategies in the field of flour	- Allocating wheat quotas to flour mills based on the quality of the flour produced **-** Aerating and storing flour in silos to reduce its moisture - Increase the extraction rate to achieve the national standard level of bran in flour	*“Flour mills receive their monthly quota from the government for producing whether high or poor-quality flour, so they don’t have any concern for being deprived, and do not make efforts to produce high-quality flour. The government should rate flour mills based on flour quality. The rating creates a competitive environment among flour mills” (Head of traditional bread bakers’ union).*
Strategies in the field of bakery	- Holding training courses for bakery workers - Improving the economic situation of bakers **-**Annual pricing of bread commensurate with inflation and its production costs - Considering non-financial penalties for offending bakers - Standardizing bread-making devices - Investing in improving the baking technology **-** Standardizing the physical space of bakeries - Ranking the bakeries and appreciating the top bakeries for producing high-quality bread	*“A bakery worker must receive practical training, get information about wheat and flour, and gain experience baking bread. Having expertise and experience in this field is a significant issue. A baker specializing in baking bread can deliver good bread to the customer and vice versa; an untrained baker cannot turn even the best flour into bread. That’s why training is so important for bakers” (Head of traditional bread bakers’ union).*
Strategies in the field of wheat-flour- bread chain management	- Assigning a specific and unified management apparatus to deal with bread issues - Strengthening the cross-sectoral cooperation to improve the quality wheat-flour- bread chain - Forming the committees to address issues related to bread quality in different provinces of the country - Providing a strategy to manage salt used in bakeries in hot weather conditions by the relevant authorities	*“You see, one person or an organization should be responsible in the field of bread, and others should cooperate with him. It should not be the case that I consider myself responsible for various issues related to bread, and another considers himself responsible. This makes a chaotic situation” (One of the key stakeholders).*
Strategies in the field of consumers	- Holding educational programs within bread-related topics	*“Culture-building activities should be implemented. Educational programs related to this issue should be broadcast on radio and television. The media should discuss the correct ways of storing and consuming bread and the health characteristics that a good quality bread should have. These are issues that need to be given much attention, especially by the Ministry of Health " (One of the key stakeholders).*

### Strategies in the field of wheat

One of the most frequently mentioned reasons for the poor bread quality was the low quality of the flour that results from the low quality of domestic wheat, especially wheat purchased by the government at a guaranteed price. Management and planning for producing high-quality wheat, industrialization of the agricultural system, observance of the correct principles of wheat planting and harvesting, and mandatory implementation of the wheat standard were suggested to improve the quality of domestic wheat. However, some suggested importing wheat from abroad and mixing it with domestic grain.

The participants suggested that the government’s price to buy grain from farmers should be based on the flour’s quality. They believed that this method would produce better quality wheat by providing greater accuracy in choosing seeds, the appropriate time for planting, fertilizing, and watering, and better management in controlling weeds and pests. Transferring grain purchases to the non-governmental sector was also suggested; due to the government not being careful enough in buying wheat.

Some participants stated that the government-guaranteed purchase made it impossible to separate the wheat according to the quality criteria. They suggested separating wheat in terms of quality in factories. They also stated that different wheat varieties could be mixed proportionally to get suitable flour with physical and chemical properties.

According to some participants, due to the limited capacity of silos, wheat is immediately milled after harvesting and immediately given to bakeries. The flour obtained is not technically usable for bread-making due to the gluten fluidity. Conserving wheat in silos for about two months improves wheat quality indicators and enhances bread quality.

### Strategies in the field of flour

Allocating wheat quotas to flour mills based on the quality of the flour produced was suggested to create a competitive environment among flour mills to make better quality flour. Other strategies offered in the study were aerating flour and then storing it in silos to reduce its moisture content and increase the extraction rate to achieve the national standard of bran in flour.

### Strategies in the field of bakeries

The lack of expertise and experience of bakery workers was considered one of the most critical barriers to improving bread quality. The participants believed that holding training courses to raise knowledge and enhance the performance of bakers in health issues and optimal conditions for preparing the dough can improve bread quality.

Some participants stated that the rising bakery costs, including water, electricity, gas, and workers’ wages, have been among the main problems for bakers in recent years. At the suggestion of the participants, improving the economic situation of the bakers through the annual pricing of bread commensurate with inflation and its production costs and considering non-financial penalties for bakers who ignore legal requirements can significantly impact improving the bread quality.

The participants, referring to the ban of baking bread on direct heat due to health problems, suggested standardizing bread-making devices, improving the baking oven, and investing in baking technology to make healthier bread. In addition, the need for standardizing the physical space of bakeries to set up a bread baking machine, store flour and bakery additives, set up a table for kneading bread dough, etc., was emphasized.

Ranking bakeries according to the quality of bread produced and appreciating the top bakeries to create competition among bakers were also suggested to improve bread quality.

### Strategies in the field of wheat-flour- bread chain management

Another issue identified in the present study was the multiplicity of responsible organizations in the bread sector and subsequent problems. Most participants pointed out that assigning a specific and unified management apparatus to deal with bread issues can be one of the most important and influential ways to improve bread quality. They believed that having a single responsible authority to address issues related to the whole wheat-flour-bread chain, from beginning to end, and the cooperation of other organizations would solve many existing problems.

Given the discussions about the need to use more salt when preparing bread dough in regions with high temperatures, the participants called for a strategy by the relevant authorities to manage salt used in bakeries in hot weather conditions.

### Strategies in the field of consumers

Some participants pointed out that consumers do not know the characteristics of good quality bread, do not know the correct principles of cooling bread after baking, and do not know how to store it at home properly. According to them, these issues might cause decreased bread quality and increased waste. Therefore, they suggested designing and implementing educational programs to raise public awareness about the characteristics of good quality bread and sound principles of storage and consumption to improve bread quality and reduce waste.

## Discussion

Data analysis showed a wide range of strategies to improve the quality of the wheat- flour- bread chain, including improving the quality of raw materials, improving the level of knowledge and skills of bakery workers, using standard bread making machines, and, most importantly, assigning a specific and unified management apparatus to deal with bread issues. Our results echo the findings of previous studies, which have shown that low-quality flour is one of the most critical barriers to improving bread quality [12]. Storing wheat in optimal storage conditions [13] and mixing different wheat cultivars with varying protein content [14] are essential for obtaining high-quality flour, as mentioned in the present study.

The study also emphasized the pivotal role of bakers in final product quality. Lack of adequate knowledge and skill in preparing and processing dough, proper baking techniques, and adopting good hygiene practices to serve customers leads to decreased bread quality and high volumes of waste [9, 15, 16]. Therefore, designing and implementing appropriate training programs for bakery works was suggested, which re-emphasizes previous studies on establishing and strengthening bakers’ training centers [9, 16, 17].

As mentioned in the study, hygienic principles are usually ignored in small bakery units, resulting in unwanted contaminants [16, 17]. Given the importance of hygiene, the participants suggested improving the hygiene conditions of bakeries and continuous and accurate monitoring of the performance of bakeries by relevant organizations.

Another issue raised was the existence of traditional ovens and non-standard bread machines in some bakeries. Use of faulty or inappropriate baking equipment can cause many defects such as changes unpleasant in texture, smell, taste, or color; the high pasting and burning rate; contamination with the fuel used to bake bread; and premature staleness [18]. Since direct heating from burning fossil fuels for baking bread poses a serious threat to human health [19], it is recommended that bread be produced by mechanized methods. In the present study, this issue was addressed, and the participants suggested investing in baking technology to improve bread quality.

The economic situation of bakers was mentioned as another factor affecting bread quality in the present study. In adverse financial status, bakers have no choice but to reduce the number of workers or use cheaper and less experienced workers, leading to a decrease in the bread quality, as reported in previous studies [20]. This issue requires the serious attention of the government and relevant officials. The ranking of bakeries based on the quality of the final product was also considered another effective way to improve bread quality. In a competitive environment, competitors seek to offer better quality products to attract customers [21].

This study mentioned the multiplicity of responsible organizations in the bread sector as an obstacle to improving bread quality. Therefore, assigning a unified management apparatus to deal with bread issues and the necessity of cooperation and coordination between organizations related to this field was one of the most effective ways to improve bread quality in the present study.

The method of storing bread at home can also effectively improve bread quality and reduce waste. Storage conditions such as temperature and humidity can significantly affect microorganisms’ retrogradation process and growth rate [22, 23]. Accordingly, in line with other studies [24], public education about the characteristics of good quality bread and sound principles of bread storage and consumption was another way to improve the quality and reduce bread waste in the present study.

In conclusion, this study revealed various stakeholders’ perspectives on strategies to improve the quality of the wheat-flour-bread chain in Iran. Accordingly, it is necessary to take specific steps to improve the quality of raw materials and factors involved in baking bread, strengthen the management and monitoring system of the wheat-flour-bread chain, and raise public awareness of storing and consuming bread properly. The results provide helpful information to guide policy decisions concerning improving wheat- flour- bread chain quality and promoting public health.

### Limitations

One of the limitations was the unwillingness of the Iranian Association of Flour Milling Industries and flour mills to participate in the research and share their experiences and information about the topic. Another limitation was that, despite obtaining national government opinions, some participants were located in Tehran, and their views could not be generalized to the rest of the nation.

## Electronic supplementary material

Below is the link to the electronic supplementary material.

Supplementary Material 1

Supplementary Material 2

## Data Availability

All data analyzed during this study are included in the article.

## Abbreviations

MAXQDA: MAX Qualitative Data Analysis.

## References

[1] Silow C, Axel C, Zannini E, Arendt EK (2016). Current status of salt reduction in bread and bakery products–a review. J Cereal Sci.

[2] Israr T, Rakha A, Sohail M, Rashid S, Shehzad A (2016). Salt reduction in baked products: Strategies and constraints. Trends Food Sci Technol.

[3] Karizaki VM (2017). Ethnic and traditional Iranian breads: different types, and historical and cultural aspects. J ethnic foods.

[4] Azadnajafabad S, Ebrahimi N, Mohammadi E, Ghasemi E, Moghaddam SS, Aminorroaya A (2021). Disparities and spatial variations of high salt intake in Iran: a subnational study of districts based on the small area estimation method. Public Health Nutr.

[5] Jafari M, Mohammadi M, Ghazizadeh H, Nakhaee N (2016). Feasibility and outcome of reducing salt in bread: a community trial in Southern Iran. Glob J Health Sci.

[6] Kurek MA, Wyrwisz J, Piwińska M, Wierzbicka A (2015). Influence of the wheat flour extraction degree in the quality of bread made with high proportions of β-glucan. Food Sci Technol.

[7] Rinaldi M, Paciulli M, Caligiani A, Sgarbi E, Cirlini M, Dall’Asta C (2015). Durum and soft wheat flours in sourdough and straight-dough bread-making. J Food Sci Technol.

[8] Różyło R, Laskowski J (2011). Predicting bread quality (bread loaf volume and crumb texture). Pol J Food Nutr Sci.

[9] Omidvar N, Aminpor A, Ghavam Sadri M, Kavian F, Rokni S, Knowledge (2007). Attitude and bread bakers in Tehran on various aspects of bread production. Iran J Nutr Food Sci.

[10] Demirtaş B, Kaya A, Dağıstan E (2018). Consumers’ bread consumption habits and waste status: Hatay/Turkey Example. Turkish J Agriculture-Food Sci Technol.

[11] Guest G, MacQueen KM, Namey EE (2012). Introduction to applied thematic analysis. Appl thematic Anal.

[12] Shahedi M. Bread losses and ways to reduce it. 1st Conference on preventing waste of national resources; Tehran2004. p. [In persian].

[13] Movahhed S, Chenarbon HA, Darabi F (2021). Assessment of storage time on dielectric constant, physicochemical and rheological properties of two wheat cultivars (Pishtaz and Hamon). J Food Meas Charact.

[14] Podolska G, Aleksandrowicz E, Szafrańska A (2020). Bread making potential of Triticum aestivum and Triticum spelta species. Open Life Sciences.

[15] Partovi R, Heidarmah F, Mohamadi M, Safari H. Investigating environment hygiene situation and quality of bread in Isfahan bakeries. 10th Seminar on Environment Hygiene; Hamedan, Iran [In persian].2007.

[16] Gholami Parizad A, Amarlooei A, Jalali Glosang A, Naseri- Far R (2005). Bread and its health problems in the bakeries of the urban areas of Ilam province, 2003–2004. J Ilam Univ Med Sci.

[17] Malacootian M, Luluei M (2003). Bread quality and health status of bakeries in Rafsanjan. J Rafsanjan Univ Med Sci.

[18] Ahrné L, Andersson C-G, Floberg P, Rosén J, Lingnert H (2007). Effect of crust temperature and water content on acrylamide formation during baking of white bread: Steam and falling temperature baking. LWT-Food Sci Technol.

[19] Mojarrad MH. Identifying the causes of bread waste in the country and ways to prevent it. Bread: technical, nutritional, health, economic and social issues. Proceedings of the Specialized Bread Summit; Tehran: National Nutrition and Food Technology Research Institute; 1995.

[20] Amini-Rarani M, Abutoraabi SH, Nosratabadi M. The Role of Social Health and Demographic Factors in Bread Quality: An Ecological Study in Isfahan, Iran. Journal of Food Quality. 2021;2021.

[21] Su H-C, Linderman K, Schroeder RG, Van de Ven AH (2014). A comparative case study of sustaining quality as a competitive advantage. J Oper Manag.

[22] Alpers T, Kerpes R, Frioli M, Nobis A, Hoi KI, Bach A (2021). Impact of Storing Condition on Staling and Microbial Spoilage Behavior of Bread and Their Contribution to Prevent Food Waste. Foods.

[23] Santos JL, Chaves RD, Sant’Ana AS (2017). Estimation of growth parameters of six different fungal species for selection of strains to be used in challenge tests of bakery products. Food bioscience.

[24] Alibeigi AH, Geravandi S, Athari Z (2011). Methods to Reduce Bread Waste among Housewives in Kermanshah. Sci J Manage Syst.

