# Supplementary material for: Strategies to improve the quality of wheat- flour- bread chain in Iran: the perspective of different stakeholders
Source: BMC Res Notes. 2022 Oct 22;15:331. doi: 10.1186/s13104-022-06225-7 (PMC9588239; doi:10.1186/s13104-022-06225-7)
Supplement: Supplementary file 1 — Supplementary Material 1 [file 13104_2022_6225_MOESM1_ESM.docx]

**Additional file 1. Semi-structured topic guides for focus groups and interviews***

| 1 | Which traditional bread (Lavash, Taftoon, Barbari, or Sangak) do you eat? |
| --- | --- |
| 2 | In your opinion, how is the quality? Why? |
| 3 | What factors affect bread quality? |
| 4 | Which organizations or institutions are responsible for improving the bread quality? |
| 5 | In your opinion, which organization or institution is better to be selected as responsible for improving the bread quality in the country? |
| 6 | What strategies do you suggest to improve the bread quality? |

***** The interview/focus group guide was designed according to the position of the stakeholders. The main questions were followed by some probe questions to acquire the required data fully.
