# Supplementary material for: Strategies to improve the quality of wheat- flour- bread chain in Iran: the perspective of different stakeholders
Source: BMC Res Notes. 2022 Oct 22;15:331. doi: 10.1186/s13104-022-06225-7 (PMC9588239; doi:10.1186/s13104-022-06225-7)
Supplement: Supplementary file 2 — Supplementary Material 2 [file 13104_2022_6225_MOESM2_ESM.docx]

**More details of sampling and design study**

Literature review and consulting with experts led to identifying four groups of stakeholders (Table 1). In total, 117 people participated in the present study. Consumer stakeholders and bakers were selected by purposive sampling from different geographical areas of Tehran, and snowball sampling was used to identify other stakeholders. Focus groups (FGs) and individual interviews were used to collect the views of consumers and other stakeholders, respectively. The duration of interviews and FGs lasted 1-2 h.

Office correspondence was made with the relevant organizations to arrange the interviews with informed and experienced persons in the field of study to collect data from key stakeholders (except for traditional bread bakers). Concerning traditional bread bakers, to achieve high diversity and variation of participants, Tehran, the capital of Iran, was geographically divided into five regions: north, south, west, east, and center, and sampling was done in such a way as to cover all geographical regions. For this purpose, one district was randomly selected from each geographical region. Four bakeries (one sangak bakery, one barbary bakery, one taftoon bakery, and one lavash bakery) were randomly chosen in that district. In each bakery, the first author interviewed the person most involved in making bread. This process continued in other geographical regions of Tehran until data saturation occurred. It should be noted that data saturation occurred in the 18^th^ interview, following which two more interviews were conducted to ensure data saturation.

To obtain additional reach data on consumer stakeholders' views, FGs were separately conducted for housewives women and employed women/ men by the main researcher and assisted by an assistant moderator. Like the bakers, sampling was done from different geographical regions of Tehran to ensure sample diversity. For housewives, one local community center in one of the districts from one geographical region belonging to Tehran Municipality’s Social and Cultural Department was randomly chosen. Housewives, who expressed an interest in participating in the study, were invited to join the study by telephone. Conducting FGs continued at other local community centers in different geographical regions of Tehran until data saturation was achieved. For employed women and men, FGs were conducted at government organizations, including universities, departments of education, and municipality councils, randomly chosen from different geographic regions of the city. Women and men working in the organizations mentioned above, with similar inclusion criteria for housewives, were invited to join the study. Ten focus groups were conducted with a maximum of nine participants per group (range = 8-10). Eight focus groups were conducted to collect data, and two more were considered to ensure no new codes and data saturation.

All interviews were conducted in a separate room using a semi-structured guide (Additional file 1) by the main researcher, and a second researcher took notes. The interview/discussion guide was designed according to the study objectives and considering the stakeholders' positions. The questions were evaluated for their content by three experts related to the research field and then pretested with some people similar to the target population before conducting interviews with the target audience. Based on their feedback, minor changes were implemented.

At the beginning of each interview session, the purpose of the meeting and the confidentiality of the acquired data was explained. Also, after ensuring the participants’ agreement with attending the meeting, all participants were asked to complete a short socio-demographic survey.
